# Whole-Genome Sequence of a Novel Species, *Mycobacterium yongonense* DSM 45126^T^

**DOI:** 10.1128/genomeA.00604-13

**Published:** 2013-08-08

**Authors:** Byoung-Jun Kim, Bo-Ram Kim, So-Young Lee, Seung-Hyeok Seok, Yoon-Hoh Kook, Bum-Joon Kim

**Affiliations:** Department of Microbiology and Immunology, Cancer Research Institute, Institute of Endemic Diseases, Seoul National University Medical Research Center (SNUMRC), Seoul National University College of Medicine, Seoul, Republic of Korea

## Abstract

Here, we report the complete genome sequence of *Mycobacterium yongonense* DSM 45126^T^, genetically closely related to the INT5 genotype of *M. intracellulare*.

## GENOME ANNOUNCEMENT

*Mycobacterium intracellulare*, a member of the *M. avium* complex, has been reported to be divided into 5 genetically distinct groups (INT1 to INT5) (1). To date, we have introduced the genome sequences of five *M. intracellulare* strains, one strain of the INT1 genotype (*M. intracellulare* MOTT-64 [GenBank number CP003324] [2]), two strains of the INT2 genotype (*M. intracellulare* ATCC 13950^T^ and MOTT-02 [GenBank numbers CP003322 and CP003323, respectively] [3, 4]), and two strains of the INT5 genotype (*M. intracellulare* MOTT-36Y and MOTT-H4Y [GenBank numbers CP003491 and AKIG00000000, respectively] [5, 6]).

Recently, we introduced a novel species, *Mycobacterium yongonense*, which is phylogenetically related to *Mycobacterium intracellulare* but has a distinct RNA polymerase β-subunit gene (*rpoB*) sequence that is identical to that of *Mycobacterium parascrofulaceum*, suggesting the acquisition of the *rpoB* gene via a potential lateral gene transfer (LGT) event (7). To gain better insight into the LGT event mechanisms in *Mycobacterium yongonense*, we have determined the complete genome sequence of *Mycobacterium yongonense* DSM 45126^T^.

The *M. yongonense* DSM 45126^T^ genome was sequenced using four types of sequencing methods—standard shotgun GS FLX pyrosequencing (770,801 reads), short paired-end GS FLX pyrosequencing (470,728 reads), shotgun clone library Sanger chemistry sequencing (11,211 reads), and fosmid library Sanger chemistry sequencing (822 reads)—to generate scaffolds containing 167 contigs. Sequencing analysis was performed at the National Instrumentation Center for Environmental Management (NICEM) (Genome Analysis Unit) at Seoul National University. A total of 1,253,562 reads were generated, with an average read length of 180 bp, yielding 225,567,303 bp sequences. This represents ~40× coverage for the estimated 5.5-Mb genome size. All the remaining gaps between contigs were completely filled by ~50-fold Solexa reads and PCR amplifications. Genome annotation was performed using the NCBI Prokaryotic Genomes Automatic Annotation Pipeline (PGAAP) (http://www.ncbi.nlm.gov/genomes/static/Pipeline.html).

Our data for the *M. yongonense* genome show it to have a circular DNA of 5,521,023 bp, a circular plasmid of 122,976 bp, and a linear plasmid of 18,089 bp. The *M. yongonense* genome contains protein-coding genes (5,222 open reading frames [ORFs]) similar to those of *M. intracellulare* ATCC 13950^T^ (5,145 ORFs) and *Mycobacterium* sp. MOTT-36Y (5,128 ORFs) and contains the same number of tRNA genes as *M. intracellulare* ATCC 13950^T^ (47 tRNA genes) but not *Mycobacterium* sp. MOTT-36Y (46 tRNA genes). The genome of *M. yongonense* DSM 45126^T^ has a G+C content of 67.95%, and the two plasmids have G+C contents of 65.92% and 66.69%. A comparison of predicted ORFs of *M. yongonense* DSM 45126^T^ with *M. intracellulare* ATCC 13950^T^ and *Mycobacterium* sp. MOTT-36Y showed that they shared 4,646 ORFs (average identity, 95.1%) and 4,932 ORFs (average identity, 96.8%), respectively. A total of 502 ORFs (9.8%) and 576 ORFs (11.0%) were specific to *M. intracellulare* ATCC 13950^T^ and *M. yongonense* DSM 45126^T^, respectively, and 351 ORFs (6.8%) and 290 ORFs (5.6%) were specific to *Mycobacterium* sp. MOTT-36Y and *M. yongonense* DSM 45126^T^, respectively.

### Nucleotide sequence accession numbers.

The whole genome sequences of *M. yongonense* DSM 45126^T^ have been deposited at GenBank under the accession numbers CP003347 (for chromosomal DNA) and JQ657805 and JQ657806 (for two plasmids).
